# Comprehension of Connectives: Development Across Primary School Age and Influencing Factors

**DOI:** 10.3389/fpsyg.2020.00814

**Published:** 2020-05-20

**Authors:** Anna Volodina, Sabine Weinert

**Affiliations:** Department of Developmental Psychology, Faculty of Human Sciences and Education, University of Bamberg, Bamberg, Germany

**Keywords:** connectives, academic language, primary school age, socioeconomic status, latent growth curve models

## Abstract

Language development is not completed when children enter primary school. As the comprehension of connectives (such as *although*, *despite*) is important for understanding and producing academic texts and, thus, relevant for school success, we investigated its development and influencing factors across primary school age on the basis of a newly developed and validated test instrument. Using a German sample of 627 students (*n* = 361 language minority learners) in primary school, results of growth curve models showed students’ initial level of the comprehension of connectives to be negatively related to its growth rate. Additional analyses revealed this association to be mainly due to parental socioeconomic status (SES) rather than students’ language background. In particular, parental SES and students’ receptive grammar impacted initial level as well as growth rate of connective comprehension. Our results point to the necessity of a continuous and early sensitization for the register of academic language especially in the group of students from a low socioeconomic background.

## Introduction

Language development further progresses after preschool age (Karmiloff-Smith, 1986, 1992), with learners expanding their proficiencies in vocabulary, grammar, and discourse through adolescence and potentially through their entire life as they move through a range of social contexts (Berman and Ravid, 2009). Connectives (e.g., *despite*, *therefore*) are cohesive devices that explicitly indicate how clauses or sentences are to be meaningfully linked (Degand and Sanders, 2002; Cain and Nash, 2011; Crosson and Lesaux, 2013a, b; Duggleby et al., 2015). In particular, the knowledge of connectives is associated with text comprehension (e.g., Crosson and Lesaux, 2013b; Duggleby et al., 2015; Kohnen and Retelsdorf, 2019), whereas the productive use of connectives is related to more complex argumentation in academic texts (Schmalhofer et al., 2002; Taylor et al., 2019). Previous studies suggest that the knowledge of connectives develops gradually and is not complete when children enter schools (e.g., Crowhurst, 1987; Cain and Nash, 2011; Knoepke et al., 2017). It is especially relevant to academic language proficiency (Schleppegrell, 2001, 2004; Barr et al., 2019). In particular, Bailey (2007, pp. 10–11) defines being academically proficient as “knowing and being able to use general and content-specific vocabulary, specialized or complex grammatical structures, and multifarious language functions and discourse structures – all for the purpose of acquiring new knowledge and skills, interacting about a topic, or imparting information to others.”

In comparison to more informal everyday language, academic language is characterized as being used in academic contexts and as consisting of more complex lexical and grammatical structures than everyday language (Cummins, 2000; Schleppegrell, 2004; Bailey, 2007). Academic language proficiency has been shown to be significantly related to academic discourses and to facilitate learning in various school subjects (Townsend et al., 2012; Haag et al., 2013; Uccelli et al., 2015; Schuth et al., 2017). Recent studies focused on the development of two facets of academic language proficiency in primary school – academic vocabulary (Volodina et al., 2020) and listening comprehension of academic language at the text level (Heppt and Stanat, in press) – and found evidence for a *Matthew effect* (Merton, 1968; Stanovich, 1986), with monolingual majority language students and students with higher socioeconomic background socioeconomic status (SES) showing a comparatively higher initial level of the aforementioned skills in academic language as well as a comparatively more rapid growth rate. To date, studies predominantly considered the comprehension of connectives using instruments measuring academic language skills cross-sectionally and mainly in secondary school children (e.g., Uccelli et al., 2015; Barr et al., 2019). However, the development of the comprehension of connectives has not yet been studied longitudinally across primary school age, which may be partly due to the lack of appropriate assessment instruments.

For the present study, a newly developed and validated test instrument for measuring the comprehension of connectives in the German language has been employed (Schuth et al., 2015; Heppt et al., 2020). The aim of this study is to contribute to a more profound understanding of the development of the comprehension of connectives in a German sample of students across primary school age and its influencing factors.

## Connectives: Definition and Characteristics

Connectives are important as they represent “a specification of the way in which what is to follow is systematically connected to what has gone before” (Halliday and Hasan, 1976, p. 227). Thus, their function is to emphasize and elucidate the logical relationship between propositions to maintain local coherence (Halliday and Hasan, 1976; Crosson and Lesaux, 2013a). Connectives are particularly important in the written register, as those who fail to note or understand them may interpret the interrelation between ideas incorrectly (Halliday and Hasan, 1976). Four major types of connectives [i.e., additive (e.g., *moreover, further, also, furthermore*), causal (e.g., *because, hence, therefore, since*), temporal (e.g., *before, next, finally, after*), and adversative (e.g., *however, although, in contrast, alternatively*)], which differ with respect to the meaning relations they signal, are generally distinguished (Celce-Murcia and Larsen-Freeman, 1983).

Connectives are an important facet of academic language (e.g., Schleppegrell, 2004; Snow and Uccelli, 2009; Barr et al., 2019), defined as “the language that is used by teachers and students for the purpose of acquiring new knowledge and skills…imparting new information, describing abstract ideas, and developing students’ conceptual understanding” (Chamot and O’Malley, 1994, p. 40). Academic language is often contrasted with more informal, interactional language (cognitive academic language proficiency vs. basic interpersonal communication skills; Cummins, 1979, 2000, 2008). Generally, the differences between academic and more informal, interactional language are regarded as a continuum, which is assumed to range from rather basic lexical and grammatical structures and a high degree of contextual embedding to a high level of complexity in vocabulary (i.e., the presence of general academic vocabulary and specialized terminology) and grammar (e.g., an extended use of prepositional phrases, nominalizations) and a low degree of contextual embedding (Gogolin, 2004; Eckhardt, 2008; Snow, 2010). Using an assessment instrument that, amongst others, measured the comprehension of connectives, Phillips Galloway and Uccelli (2019) showed the test scores to be positively associated with the quality of science summary writing in students in Grades 4–8, predicting unique variance over and above students’ demographic characteristics (gender, language status, SES, race/ethnicity, and special education status) and their reading comprehension.

Connectives vary in their complexity and frequency (Crosson and Lesaux, 2013a). Generally, children produce a range of connectives already by the age of 5 (Kail and Weissenborn, 1991; Spooren and Sanders, 2008), and their acquisition develops gradually. Connectives expressing additive relations (e.g., *and*) are generally acquired first. Then, connectives which express temporal, causal, and adversative relations are acquired (Bloom et al., 1980; Goldman and Murray, 1992). The sequence of the acquisition of connectives is associated with the cognitive complexity of the specific coherence relation (Sanders et al., 1992) and is guided by such parameters as polarity (positive vs. negative) and the strength of relation (e.g., additive vs. causal) (Spooren and Sanders, 2008). In addition, the degree of syntactic complexity is related to the relative order in which various connectives, which express (nearly) the same coherence relation, emerge. Furthermore, the degree of syntactic complexity is associated with the order in which various uses of one and the same connectives emerge (Eversl-Vermeul and Sanders, 2009). However, production of connectives does not ensure that their meaning is fully understood (Crosson et al., 2008). Previous research found the comprehension both of connectives and of coherence relations to still develop during primary school years (McClure and Geva, 1983; Cain et al., 2005; Cain and Nash, 2011; Pyykkönen and Järvikivi, 2012).

In particular, research findings indicate that students in primary school still show pronounced restrictions in processing negative-causal connectives. Dragon et al. (2015) found German students in Grades 2 and 3 to hardly process connectives in negative-causal sentence pairs (connected, e.g., by *although*, *despite*) compared to their comprehension of positive-causal sentence pairs (connected, e.g., by *therefore*, *thus*). When confronted with a task to indicate whether sentence pairs made sense, primary school students systematically rejected negative-causal sentence pairs that were semantically coherent due to the connective and accepted negative-causal sentence pairs that were incoherent due to the connective. Dragon et al. (2015) suggested that most primary school students ignored the negative-causal connectives and made judgments of the sentence pairs only based on the semantic scenario and situational compatibility, while ignoring the meaning of the connectives. In a similar vein, drawing on a sample of primary school students in Grades 1–4, Knoepke et al. (2017) showed that students’ comprehension of negative-causal coherence relations develops through primary school age. Cain and Nash (2011) found 10-year-old students to be more accurate on adversative and temporal connectives than 8-year-old students in a cloze task, with both age groups showing significant differences from the adult level of performance. However, when confronted with the task to rate the sense of two-clause sentences linked by connectives, 10-year-olds were better at discriminating between clauses linked by appropriate and inappropriate connectives than were 8-year-olds and differed from adults only with regard to the temporal connectives. Pyykkönen and Järvikivi (2012) showed that 12-year-old students experienced difficulties in understanding temporal relations when the connective used (*after*, *before*) implies a reversed order of the related propositions with respect to the chronological order of events in the world. McClure and Geva (1983) also demonstrated that students in Grade 4 struggle with understanding the differences of focus between the connectives *although* and *but* as a function of their syntactic position (i.e., initial or medial) within the sentence. Furthermore, there is a difference in the age of acquisition between connectives that are typically used in informal oral communication compared to those predominantly used in the written register (e.g., Crosson et al., 2008). In particular, in recent studies, Zufferey and Gygax (2020a, b) found adolescents and even adults to have difficulties in understanding connectives typical for the written register in comparison to those frequently used in everyday communication; the former ability was highly associated with exposure to print. In addition, in the adult sample, the ability to understand connectives typical for the written register was also associated with their general grammatical skills (Zufferey and Gygax, 2020b).

Although children’s knowledge of connectives is still developing during primary school age, there is some evidence that primary school students have begun to profit from the information provided by connectives to support comprehension during the reading process (Cain and Nash, 2011). Furthermore, students’ knowledge of connectives is associated with school performance in primary school (Heppt et al., 2020; Volodina et al., unpublished).

## Influencing Factors on the Acquisition of Knowledge of Connectives

Comprehension of connectives is associated with various potentially influencing factors at the individual level. One of these is the families’ language background. According to Cummins (1981) and Hakuta et al. (2000), the acquisition of academic language takes more time for children whose native language is not the same as that in which the schooling takes place. These children may benefit less from learning opportunities at school, as they often don’t speak the language of schooling at home (Scheele et al., 2010). In particular, results of studies by Verhoeven (2000) and Droop and Verhoeven (2003) showed that, in the Netherlands, language minority students from low-SES families experienced more difficulties identifying connectives that meaningfully link two clauses or sentences compared to their monolingual peers. Crosson and Lesaux (2013b) reported significantly higher performance of monolingual English-speaking students in Grade 5 on tasks on the comprehension of connectives compared to language minority learners. In their study, both groups of children were from low-SES families and were demographically at risk for difficulties in literacy tasks. Furthermore, Welie et al. (2017) found monolingual Dutch eighth graders to outperform language minority learners on knowledge of connectives; in the same vein, Kohnen and Retelsdorf (2019) recently documented an advanced comprehension of connectives in monolingual German students compared to language minority students in Grade 9.

In addition to language background, the bioecological model of development (Bronfenbrenner, 1979; Bronfenbrenner and Morris, 1998) underlines socioeconomic background (SES) as an important source of environmental effects on language development. Considering various indicators of SES, especially parental education is significantly associated with children’s language development (Hoff, 2006). In particular, children in families with higher educational qualifications are presented with a high quantity of words and lexically diverse vocabulary (Hart and Risley, 1992; Hoff, 2006; Golinkoff et al., 2019). Further, highly educated parents have been suggested to often provide their children with joint activities that are considered as cognitively stimulating and that have been shown to be associated with children’s language acquisition, including progression in vocabulary and grammar (e.g., Hart and Risley, 1992; Harding et al., 2015). In general, when compared to parents with a lower SES, parents with a comparatively higher SES tend to use more complex sentence structures and higher numbers of nominal phrases per sentence (Huttenlocher et al., 2002, 2010). In addition, the early association between social disparities and child grammar at age 3 has been shown to remain constant over 2 years of preschool education and to predict later school learning (Weinert et al., 2010; Kotzerke et al., 2013; Weinert and Ebert, 2013). Furthermore, Uccelli et al. (2019) found that the families’ SES, the amount of relatively decontextualized parent talk, and children’s vocabulary at the age of 30 months all independently contributed to students’ academic language proficiency in Grade 7. Additionally, according to the family investment model (Conger and Donnellan, 2007), higher-SES families are more prosperous and, thus, have a possibility to provide their children with better material resources at home (e.g., books, computers).

In line with some of the aforementioned assumptions, constructivists consider the variations in input to be critical to language outcomes in children (e.g., Tomasello, 2000; Saffran, 2001). Having acquired some basic syntactical skills, children can use them to bootstrap the meanings of more complex syntactical structures (Naigles and Hoff-Ginsberg, 1995). In addition, the acquisition of connectives has been suggested to be related to listening comprehension and vocabulary knowledge, and respective associations have been shown in a sample of language minority learners from Spanish-dominant homes and low SES in Grade 4 when word reading skills were controlled for (Crosson et al., 2008). However, precursors of academic language (e.g., the ability to understand rare words that have a special technical meaning or that explicitly connect sentences and ideas) are developed before school entry in rich home learning environments (e.g., Leseman and de Jong, 1998; Leseman et al., 2007; Aarts et al., 2011), leading to an advanced awareness of the different language choices and registers that are effective for taking part in various schooling contexts (Comber and Cormack, 1997). As already mentioned, parents differ widely in the activities they perform with their children. According to Sénéchal (2006), early literacy experiences at home (e.g., storybook exposure, parents’ teaching of literacy) are strongly associated with vocabulary knowledge in kindergarten children and students’ reading attainment at Grade 4. In a similar vein, Lawson (2012) found parental involvement in reading aloud to their children to significantly predict students’ later academic achievement.

To sum up, to date and despite their suggested and cross-sectionally demonstrated importance, only a small number of studies addressed the comprehension of connectives in primary school age (e.g., Droop and Verhoeven, 2003; Dragon et al., 2015; Knoepke et al., 2017) and traced their developmental progress across grades. Furthermore, although some studies which compared the comprehension of connectives in monolinguals and language minority students suggest that connectives are particularly challenging for language minority students in the primary and middle school grades (e.g., McClure and Steffensen, 1985; Verhoeven, 2000; Droop and Verhoeven, 2003; Crosson et al., 2008), these studies mainly included language minority students from low-SES families (e.g., Droop and Verhoeven, 2003; Crosson and Lesaux, 2013b). Finally, the role of various influencing factors on the comprehension of connectives has rarely been examined comprehensively.

## Research Questions

In our study, the following research questions were addressed: *How does the comprehension of connectives develop across primary school age? Which factors affect the initial level and the growth rate of the comprehension of connectives?*

Drawing on the theoretical assumptions and empirical evidence, we expect students’ comprehension of connectives to increase from Grade 2 to Grade 4. In line with the assumptions of Cummins (1981), previous results on the comprehension of connectives in monolingual and language minority students (e.g., Droop and Verhoeven, 2003; Kohnen and Retelsdorf, 2019), and studies on the development of other facets of academic language in primary school age (e.g., Volodina et al., 2020; Heppt and Stanat, in press), we expect students from higher-SES families to outperform students from lower-SES families at least in the initial level and potentially also in growth rate of the comprehension of connectives. Comparatively higher growth rates are suggested by the fact that restricted grammatical skills (e.g., Huttenlocher et al., 2010; Weinert and Ebert, 2013) and less stimulation of academic language at home (Leseman et al., 2007; Scheele et al., 2010) might lead to a *Matthew effect* [i.e., children from higher-SES families who start out with advanced language skills and more accelerated connective comprehension and who experience higher literacy stimulation and promotion of academic language at home might progress faster compared to low-SES children (Volodina et al., 2020)]. However, it has also been argued that schooling might compensate for restricted language input and less literacy stimulation at home by presenting all students with the same chances to acquire academic language skills (Heyns, 1978; Alexander et al., 2001, 2007). This should lead to comparable growth rates or even to compensatory effects as all children now get access to more complex grammatical structures and connectives (Heyns, 1978). In addition, based on existing empirical evidence, it can be assumed that monolingual students will outperform language minority learners. Note that, in contrast to several previous studies (e.g., Droop and Verhoeven, 2003; Crosson and Lesaux, 2013b), our study includes monolingual and language minority learners from a broad range of socioeconomic backgrounds. Thus, it is an open question whether language minority students do have more difficulties in acquiring more complex connectives compared to monolingual children (implying a Matthew effect) or whether schooling leads to a compensatory effect or at least comparable growth patterns in both monolingual and multilingual children. Furthermore, in line with previous assumptions and findings (e.g., Crosson et al., 2008; Lawson, 2012; Harding et al., 2015), we expect to find an effect of joint preschool activities as well as of students’ general vocabulary and grammatical skills on the initial level and on the development of the comprehension of connectives.

## Materials And Methods

### Participants

Data stemmed from the project “Academic language proficiency (BiSpra II): Language demands, language processing and diagnostics” (German: *Bildungssprachliche Kompetenzen: Anforderungen, Sprachverarbeitung, und Diagnostik*; cf. Weinert et al., 2017, 2019), which investigated the impact, interrelation, and development of various facets of academic language comprehension. The project included two cohorts starting in Grades 2 and 3 with a total of 546 and 599 students, respectively. The data were collected in German primary schools. The selected schools had a high proportion of language minority students, since the project focused on students with either mono- or non-monolingual German-language background. Participation in the study was voluntary for both schools and students, and parents had given their written consent for their children to participate. In the present investigation, the subsample of students who were presented with a test on the comprehension of connectives at least at one measurement time point was used (*N* = 627 students from 42 classes in 21 schools), as longitudinal methods do not require full information across waves (Singer and Willett, 2003). At T1 (June 2014), 304 students were in Grade 2 and 323 students in Grade 3. Within a multicohort sequence design (for the linking of the cohorts, see below), these students were again tested 1 year later (T2; June 2015). Attrition rates across measurement points were 12.4% (*n* = 37) in Grade 3 and 4.5% (*n* = 14) in Grade 4, respectively. No significant differences (*p* > 0.05) were found between students who remained in the sample and those who dropped out in terms of their sociodemographic and personal characteristics [i.e., age, gender, language spoken at home, number of books at home, highest International Socio-Economic Index (HISEI), and parental education] or performance measures (receptive vocabulary, receptive grammar, and the comprehension of connectives at first measurement point). Students also filled in a short questionnaire including questions on their demographic background. Additionally, parents completed a short questionnaire including questions on the SES of the family.

### Measures

All tests used in the study were scaled using the one-parameter Rasch model (Embretson and Reise, 2000) and the Conquest 4 software (Adams et al., 2016).

#### Comprehension of Connectives

To assess the comprehension of connectives across grades, a scale (Heppt et al., 2020) was developed based on an examination of two large language corpora. The corpus *ChildLex3* (ca. 10 million tokens, 180,000 types; Schroeder et al., 2014) contains child-directed speech, whereas the corpus *DLex2* (about 100 million tokens, 2.3 million types; Heister et al., 2011) refers to adult-related speech. In particular, *DLex2* includes texts from predominantly written adult-directed sources (e.g., science texts) and, thus, may be considered as the corpus with a more sophisticated and formal language compared to the *ChildLex3* corpus. To select appropriate connectives, the frequency of occurrence of different connectives was analyzed. Based on these analyses, connectives that occurred more often in the *DLex2* corpus were chosen for the test [see Schuth et al. (2015) for a detailed description of scale development]. Types of connectives and their position within the test sentences were systematically varied. In particular, five different types of connectives (temporal, concessive, causal, conditional, modal) were included in the scale. During test development, different tasks and item formats were constructed and extensively tested. The results showed that asking students to select the appropriate connective out of four options was most suitable for assessing students’ comprehension of connectives in primary school (Schuth et al., 2015). Items for the final version of the scale were selected based on their difficulty, discrimination, and correlation with the overall test score (Schuth et al., 2015). Thus, in the present study, students were presented with sentences/sentence pairs and had to choose one out of four connectives that appropriately links two parts (22 items with a total of 39 connectives, with several connectives being used both as a target word and as a distractor). The construction of distractors followed a structured principle: For each item, two distractors were semantically incorrect but grammatically correct connectives, whereas one distractor comprised a connective that is semantically related to the correct choice but does not fit into the sentence grammatically. Sentence content referred to children’s everyday activities in school and recreational time, and attention was paid to not addressing any topics that might be known only by a minority of students.

In addition to the printed test booklet, all items were also orally presented via audio CDs to minimize a potential influence of students’ reading competence.

Example: *“Der Ausflug hat (aufgrund/trotz/jedoch/infolge) einer Regenwarnung stattgefunden.” [The trip took place (due to/despite/however/because of) the rain warning.]*

All items had very good fit statistics (see Heppt et al., 2019). The scale is significantly associated with other facets of academic language (e.g., academic vocabulary) (Heppt et al., 2020). Furthermore, it correlates significantly with school grades in reading, writing, mathematics, and social studies across primary school grades (Heppt et al., 2020).

Longitudinal scaling has been performed using the mean–mean linking method (Kolen and Brennan, 2004) based on an anchor-item design^1^. All the premises (e.g., one-dimensionality of instruments at all measurement points, measurement invariance between groups of students) have carefully been examined before conducting mean–mean linking. Both cohorts of students in Grade 3 (i.e., *N* = 627) were scaled simultaneously, as they did not differ with respect to age, gender, family language, number of books at home, SES of the family, and parental education (all *p* > 0.05). By linking the two cohorts, the design resulted in a three-measurement-point longitudinal design from Grade 2 through Grade 4 (cohort sequential design; Meredith and Tisak, 1990). In the longitudinal sample, the weighted likelihood estimate (WLE) reliabilities of the test on the comprehension of connectives were 0.78, 0.72, and 0.72 in Grades 2, 3, and 4, respectively.

#### General Language Skills

Students’ general language skills in the German language were measured by tests on receptive vocabulary and grammar; both were assessed at the first measurement point.

#### Receptive Vocabulary

We used a German research version of the *Peabody Picture Vocabulary Test* (PPVT; Bulheller and Häcker, 2003; Dunn and Dunn, 2003) to assess students’ general vocabulary comprehension. The test was shortened to 40 items and adapted for assessment in a classroom setting. For each item, students had to choose one picture out of four that best matched a given word. The words were orally presented via audio CDs. Test reliability was high (α = 0.87).

#### Receptive Grammar

Students’ sentence comprehension was assessed using a shortened German version of the *Test for Reception of Grammar* (TROG-D; Bishop, 1989; Fox, 2007). The test version consisted of 34 items that required students to select one picture out of four that best corresponded to an orally presented sentence [e.g., “*Weder der Hund noch der Ball ist weiß” (“Neither the dog nor the ball is white”*)]. Analogous to the receptive vocabulary measure, the test was adapted for a group setting, and all sentences were played from CD. TROG’s reliability was high (α = 0.82).

#### Language Background

Students were asked which language they speak at home. For all analyses, we used a dichotomous variable for students’ language background (1 = *monolingual German*, 0 = *non-monolingual German*). In our sample, 42.4% of the students were monolingual German. The group of language minority learners included students who spoke German and at least one other language at home (77.5%) and those who did not speak German at all in their homes (22.5%). In the group of language minority students, Turkish (17.2%), Russian (15.2%), and Arabic (11.9%) were the most spoken foreign languages at home.

#### Gender and Age

In the student questionnaire, students were asked to indicate whether they are a boy or a girl (dichotomous variable; 1 = *boy*, 2 = *girl*). In addition, students provided information on their age in years.

#### Parental Education

As an indicator of parental education, we translated the educational qualification of each parent into accumulated years of education (OECD, 2009) and subsequently used the highest number of years of education of the parents in our analyses. For additional analyses (see below), we classified parental education into low, medium, and high levels. Low educational level corresponds to levels 1 and 2 (primary education and low and intermediate secondary education), medium to levels 3A and 5B (upper secondary education and first stage of tertiary education not leading directly to an advanced research qualification), and high to levels 5A/6 (university), considered in the International Classification of Education (ISCED).

#### Parental Occupation

Information on parents’ current occupation was coded according to the International Standard Classification of Occupations (ISCO-08; International Labour Office, 2012) and subsequently transformed into the International Socio-Economic Index (ISEI; Ganzeboom et al., 1992). In our analyses, we used the HISEI of both parents as an indicator of children’s SES.

#### Cultural Resources

Parents reported the number of books at home on a five-point scale (1 = *0–10 books*, 5 = *more than 200 books*). In case this information was missing in the parental questionnaire, we used the information available in the students’ questionnaire.

For our analyses, we combined parental education, HISEI, and cultural resources into a latent variable as an indicator of SES. With three indicators, the model was saturated [*df* = 0, comparative fit index (CFI) = 1.00, Tucker–Lewis index (TLI) = 1.00, root mean square error of approximation (RMSEA) = 0.00, standardized root mean square residual (SRMR) = 0.00].

#### Parents’ Joint Activities With the Child

Parents were asked to report on their joint activities with their child before school entry using a scale from the Progress in International Reading Literacy Study (PIRLS) 2006 (Bos et al., 2010). The scale consists of 10 items and includes such activities as to read aloud to the child, to instruct the child to write letters, or to play word games, with response options ranging from 1 = *never or almost never* to 3 = *often*. The fit of a one-factor model was supported by the common indices including the CFI, the TLI, the RMSEA, and the SRMR (Marsh, 2007): χ^2^ (*df* = 32) = 63.80, *p* < 0.05, CFI = 0.964, TLI = 0.949, RMSEA = 0.046, and SRMR = 0.038. The standardized factor loadings were all above 0.3 and statistically significant (*p* < 0.001), suggesting that the indicators were sufficiently related to their purported latent factor (Brown, 2006). The scale had high reliability (α = 0.80).

### Statistical Procedures

#### Latent Growth Curve Models

With the aim to examine the effects of the potential predictors on the development of comprehension of connectives across primary school, latent linear growth curve models with three repeated measurement points were computed in the M*plus* 8.3 software (Muthén and Muthén, 1998–2017). In all analyses, age at assessment was treated as a time-varying predictor.

Initially, a separate latent linear growth curve model was conducted to test for differences in (a) starting level (intercept) and (b) growth (slope) of the comprehension of connectives for the overall sample and then depending on the children’s language spoken at home. In the first step, we specified a linear growth model. In the second step, a model that was able to capture any shape of change was specified by constraining the first loading on the growth factor to 0 and the last loading to 1, with the loading to T2 being freely estimated (e.g., Bollen and Curran, 2006).

Model fit was assessed by means of the chi-square (*χ^2^*) goodness-of-fit statistic. As this statistic is known to be highly sensitive to sample size (e.g., Marsh et al., 2004), we consulted several commonly used and recommended descriptive measures of model fit: the SRMR, the RMSEA, the CFI, and the TLI. CFI and TLI values greater than 0.90 or 0.95, SRMR values lower than 0.08 or 0.10, and RMSEA values lower than 0.05, 0.06, or 0.08 are typically considered indicative of a good model fit (Marsh, 2007; West et al., 2012).

We then used a two-step strategy with the aim to examine the effects of predictors on the comprehension of connectives. In the first step, each predictor was individually entered into a simple conditional model with the aim to evaluate its association with the initial level and growth factors. In the next step, models were specified for all students with a stepwise procedure to evaluate the relative weight of different predictor variables for the initial level and growth factors (full conditional models). Gender and language spoken at home were considered first (Model 1). Then, an indicator of SES (i.e., a measure which consisted of parental education, HISEI, and number of books at home) was included (Model 2) as a distal status indicator. In the next steps, joint preschool activities (Model 3) were added into the model as process indicators of the home (learning) environment, and, finally, students’ receptive vocabulary (PPVT) and grammar (TROG) scores (Model 4) were added into the model as indicators of child’s general language skills.

We *z*-standardized all continuous variables in the total sample prior to the analyses in order to facilitate the interpretation of results. The data have a nested structure, with students being nested in school classes. Thus, standard errors adjusted for the multilevel structure of the data were estimated by applying the *complex sample option* of the M*plus* 8.3 software (Muthén and Muthén, 1998–2017).

#### Missing Data

The estimation of the WLEs has been done for the students who completed the scale on the comprehension of connectives at least at one measurement time point. There were missing values in parental education and HISEI in the present data set (approx. 34.0%). Furthermore, PPVT and TROG scores were missing by design (approx. 46.0%). We used multiple imputation (Enders, 2010) as implemented in the MICE package (*Multiple Imputation by Chained Equations*; van Buuren and Groothuis-Oudshoorn, 2011) in R (100 data sets) to account for missing information in background variables and for WLE scores of students who did not complete the scale on the comprehension of connectives in Grade 2 or 4 (the cohort sequential design; see linking of the two cohorts). The results of subsequent analyses with 100 imputed data sets were subsequently combined in M*plus* 8.3 using Rubin’s (1987) formulas^2^.

## Results

### Descriptive Results

Table 1 presents the means, standard deviations, and correlations of the variables under study associated with the comprehension of connectives in Grades 2, 3, and 4. The mean test scores for the comprehension of connectives increased from grade to grade [9.80 (*SD* = 4.30) in Grade 2, 13.11 (*SD* = 4.94) in Grade 3, and 14.65 (*SD* = 4.51) in Grade 4; see Table 1 for mean WLE scores]. Students’ language spoken at home showed small positive correlations with scores on the comprehension of connectives. SES and joint preschool activities correlated significantly positively with students’ scores on the test on the comprehension of connectives. PPVT and TROG showed significant medium to high positive correlations with the comprehension of connectives in Grades 2–4.

**TABLE 1:** Descriptive statistics and correlations for the overall sample.

	**Variable**	***M***	***SD***	**[1]**	**[2]**	**[3]**	**[4]**	**[5]**	**[6]**	**[7]**	**[8]**
[1]	WLE Grade 2	0.07	1.41								
[2]	WLE Grade 3	1.30	1.33	0.53**							
[3]	WLE Grade 4	1.83	1.43	0.34**	0.64**						
[4]	Gender	1.51	0.50	0.03	0.07	0.06					
[5]	Family language	0.42	0.49	0.14**	0.17**	0.14**	−0.01				
[6]	SES	0.00	1.01	0.46**	0.28**	0.35**	0.03	0.30**			
[7]	Preschool activities	2.35	0.39	0.20**	0.23**	0.24**	0.04	0.16**	0.32**		
[8]	Receptive vocabulary	0.11	0.93	0.29**	0.50**	0.54**	0.03	0.16**	0.44**	0.19**	
[9]	Receptive grammar	0.08	0.70	0.23**	0.43**	0.39**	−0.01	0.26**	0.41**	0.18**	0.62**

N = 627. WLE = weighted likelihood estimate of the test on comprehension of connectives. SES = socioeconomic status of the family (an average from z-standardized parental education; highest International Socio-Economic Index of Occupational Status, and number of books at home). Gender is coded as 1 = boy, 2 = girl. **p < 0.01.

Across grades, there was a slightly higher preference for the semantically related distractors compared to the semantically inadequate though grammatically correct ones. Furthermore, monolingual German students selected semantically related distractors more frequently compared to language minority learners (*p* < 0.05). When comparing different types of connectives, data show the following pattern: When considering German monolinguals and language minority learners separately, causal connectives were significantly more difficult than concessive and temporal connectives across grades (*p* < 0.05). Furthermore, across grades, concessive connectives were significantly easier than temporal ones in the overall sample (*p* < 0.05). Yet, in Grades 2 and 3, this only held for language minority learners (*p* < 0.05), whereas no difference in difficulty was observed in the group of monolingual German students. In Grade 4, the comprehension of concessive and temporal connectives did not differ in either German monolinguals or language minority learners (all *p* > 0.05).

### Comprehension of Connectives: Differential Growth According to Language Background?

We first specified growth curve models for the overall sample. A linear growth curve model did not show good fit of data (CFI = 0.567, TLI = 0.257, RMSEA = 0.252, SRMR = 0.046). The model with a freely estimated shape of change showed good fit of data with CFI = 1.000, TLI = 1.000, RMSEA = 0.000, and SRMR = 0.000 and, in consequence, was considered for further analyses. In this model, the mean intercept was 0.06, *p* = 0.649 (variance intercept 1.67, *p* = 0.000), the mean slope 1.76 (variance slope = 1.74, *p* = 0.000), and the free loading 0.70, *p* = 0.000. Intercept and slope correlated negatively with each other (*r* = -0.58, *p* = 0.000). Thus, the initial level of students’ comprehension of connectives was negatively related to its growth.

In the next step, growth curve models for German monolinguals and language minority learners were specified. The two-group model with a freely estimated shape of change showed good fit of data with CFI = 0.996, TLI = 1.010, RMSEA = 0.012, and SRMR = 0.004 (again, a linear growth curve model did not fit the data well: CFI = 0.541, TLI = 0.213, RMSEA = 0.268, SRMR = 0.048). Language minority students differed in their initial level of performance from German monolinguals [*b* = -0.09, *p* = 0.551 vs. *b* = 0.28, *p* = 0.058; Wald-χ^2^ = 7.109 (*df* = 1), *p* = 0.008]. However, the performance gap between language minority students and German monolinguals did not change across grades [*b* = 1.53, *p* = 0.000 vs. *b* = 1.75, *p* = 0.000; Wald-χ^2^ = 0.001 (*df* = 1), *p* = 0.974]. The slope and the intercept correlated negatively within both the group of German monolinguals and the group of language minority students (*r* = -0.54, *p* = 0.000, and *r* = -0.61, *p* = 0.000, respectively).

### Prediction of the Initial Level and Growth of the Comprehension of Connectives

The results of simple conditional models for the initial level and growth factors (see Table 2) showed significant positive effects of SES, receptive grammar, and receptive vocabulary, with the explained variance of the initial level of comprehending connectives ranging between 7% and 10% for each of these variables and between 4% and 8% of the growth of the comprehension of connectives. Gender was neither predictive for the intercept nor for the growth of the comprehension of connectives. Family language and joint preschool activities significantly predicted the initial level of the comprehension of connectives (2% and 6% of explained variance, respectively) though not the growth rate.

**TABLE 2:** Simple conditional models for comprehension of connectives.

**Variable**	**Predicting intercept**				**Predicting growth**			
	**Estimate**	***SE*^a^**	***p***	***R*^2^**	**Estimate**	***SE*^a^**	***p***	***R*^2^**
Gender	0.03	0.05	0.496	0.00	0.04	0.06	0.451	0.00
Family language	0.41	0.14	0.004	0.02	0.02	0.15	0.882	0.00
SES	0.53	0.16	0.001	0.07	0.37	0.18	0.044	0.04
Preschool activities	0.56	0.20	0.006	0.06	0.09	0.22	0.672	0.01
Receptive grammar	0.44	0.09	0.000	0.10	0.39	0.11	0.000	0.08
Receptive vocabulary	0.47	0.13	0.000	0.07	0.39	0.16	0.012	0.05

**N = 627. ^*a*^Corrected standard errors for nested data. Estimate = unstandardized estimate. SES = socioeconomic status of the family. Gender is coded as 1 = boy, 2 = girl. Family language is coded as 0 = non-monolingual German, 1 = monolingual German.**

The full conditional models which considered all predictors simultaneously in a stepwise fashion (see Table 3A) underline the significant effect of family language on the initial level of the comprehension of connectives (explained variance together with gender as control variable: 3%); an additional 4% is explained by SES (Models 1 and 2). Effects of family language remained fairly constant, whereas the effect of SES was reduced when controlling for joint preschool activities (Model 3). The inclusion of receptive vocabulary and grammar in Model 4 led to a significant increase of explained variance (4%) and a reduction of the (now non-significant) effects of family language and SES. Interestingly, only the effect of receptive grammar but not that of receptive vocabulary on the comprehension of connectives was significant within the full conditional model.

**TABLE 3A:** Full conditional models predicting intercept in the overall sample.

**Variable**	**Model 1**			**Model 2**			**Model 3**			**Model 4**		
	**Estimate**	***SE*^a^**	***p***	**Estimate**	***SE*^a^**	***p***	**Estimate**	***SE*^a^**	***p***	**Estimate**	***SE*^a^**	***p***
Gender	0.09	0.12	0.466	0.07	0.12	0.529	0.06	0.12	0.591	0.05	0.11	0.648
Family language	0.41	0.14	0.004	0.26	0.13	0.045	0.24	0.13	0.061	0.18	0.13	0.171
SES				0.46	0.16	0.003	0.36	0.18	0.048	0.16	0.18	0.397
Preschool activities							0.34	0.22	0.124	0.34	0.22	0.131
Receptive grammar										0.32	0.14	0.021
Receptive vocabulary										0.07	0.20	0.719
*R*^2^	0.026			0.068			0.091			0.132		
Δ *R*^2^				0.042**			0.023**			0.041**		

N = 627. ^*a*^Corrected standard errors for nested data. Estimate = unstandardized estimate. SES = socioeconomic status. Gender is coded as 1 = boy, 2 = girl. Family language is coded as 0 = non-monolingual German, 1 = monolingual German. **p < 0.01.

**TABLE 3B:** Full conditional models predicting slope in the overall sample.

**Variable**	**Model 1**			**Model 2**			**Model 3**			**Model 4**		
	**Estimate**	***SE*^a^**	***p***	**Estimate**	***SE*^a^**	***p***	**Estimate**	***SE*^a^**	***p***	**Estimate**	***SE*^a^**	***p***
Gender	0.11	0.15	0.451	0.10	0.15	0.493	0.10	0.15	0.485	0.09	0.14	0.514
Family language	0.02	0.16	0.876	−0.10	0.16	0.547	−0.09	0.16	0.573	−0.16	0.16	0.312
SES				0.39	0.19	0.040	0.40	0.21	0.058	0.19	0.22	0.402
Preschool activities							−0.09	0.25	0.716	−0.09	0.25	0.702
Receptive grammar										0.32	0.17	0.059
Receptive vocabulary										0.11	0.25	0.650
*R*^2^	0.004			0.047			0.052			0.103		
Δ *R*^2^				0.043**			0.005			0.051**		

N = 627. ^*a*^Corrected standard errors for nested data. Estimate = unstandardized estimate. SES = socioeconomic status. Gender is coded as 1 = boy, 2 = girl. Family language is coded as 0 = non-monolingual German, 1 = monolingual German. **p < 0.01.

The results for the slope are shown in Table 3B. In Model 1, family language was not predictive of the growth of the comprehension of connectives. The inclusion of SES (Model 2) led to a significant increase of variance explained by the model (4%). Effects of SES remained fairly constant even when controlling for joint preschool activities (Model 3), which did not affect growth significantly within the full model. The inclusion of receptive grammar and vocabulary in Model 4 led to a significant increase of explained variance (5%) and mediated the effect of SES. Again, only receptive grammar, though not vocabulary, showed a marginally significant effect on the growth of the comprehension of connectives within the full conditional model.

### *Post hoc* Analyses

**FIGURE 1:**
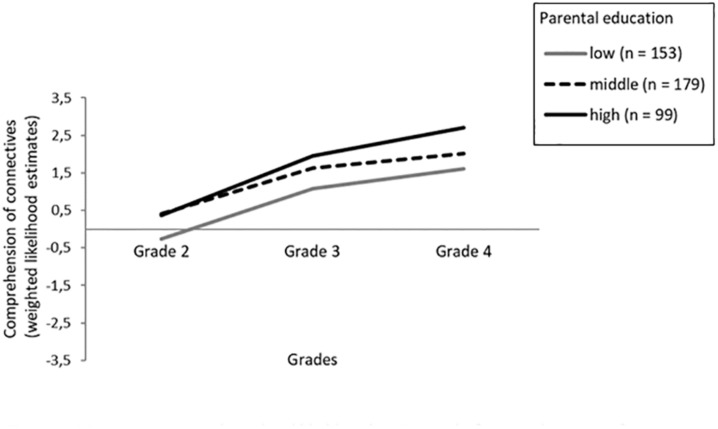
Mean test scores (weighted likelihood estimates) of comprehension of connectives differentiated for Grade and the highest parental education (overall sample).

**FIGURE 2:**
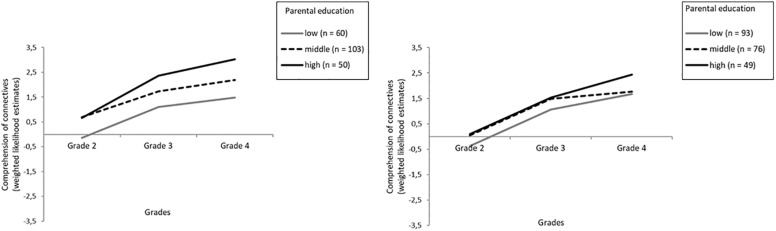
Mean test scores (weighted likelihood estimates) of comprehension of connectives differentiated for Grade and the highest parental education (sample of German monolinguals on the left and sample of language minority students on the right).

To better understand the relation and meaning of the negative correlation between slope and intercept and the effects of SES, we performed descriptive analyses of the WLEs of the comprehension of connectives by parental education, as parental education is considered to be the most stable measure of SES over the child’s life course (Bradbury et al., 2015) and can be categorized in line with established classification systems (i.e., low, middle, and high). Alongside differences due to language background, differences based on SES are often found for both general and academic language skills (e.g., Hoff, 2006; Huttenlocher et al., 2010). Figure 1 shows the means of WLEs for the comprehension of connectives for the overall sample by the highest parental education. As shown in Figure 1, whereas mean WLEs are quite similar for students whose parents have middle or high levels of education in Grade 2 (*p* > 0.05), all three groups differ in their mean WLEs in Grades 3 and 4 (*p* < 0.01). In particular, students whose parents have a medium level of education become more similar to students from low-education homes (*p* > 0.05). This pattern was especially pronounced in the group of language minority learners (see Figure 2).

## Discussion

Connectives are an important facet of academic language which has been suggested to be highly relevant to school success (e.g., Schleppegrell, 2004; Barr et al., 2019). However, to date, scarce research exists on its development. In our study, we traced the development of the comprehension of connectives across primary school age with a newly developed and validated test instrument. Our results show that the comprehension of connectives increases from Grade 2 to Grade 4, supporting previous studies which found the comprehension of connectives to still improve after children have entered school (e.g., Dragon et al., 2015; Knoepke et al., 2017). With respect to socioeconomic and language background, we find an effect of SES on the intercept as well as on the growth of comprehension of connectives across primary school; further, language minority learners showed less proficiency in Grade 2 but comparable growth rates to monolingual German students. Joint activities promoting language development added explained variance but did not account for the SES effects. However, the effects of SES became non-significant when including general language skills into the model. Receptive grammar turned out to be comparatively the best predictor of the intercept and growth of connective comprehension.

Thus, our results show that students from families with higher socioeconomic and educational status not only start with advanced skills but also exhibit comparatively enhanced progress. However, in addition, we found a negative correlation between the intercept and slope, indicating that at least some students with lower proficiency in Grade 2 show comparatively higher growth rates and thus seem to catch up, or that some of the students with comparatively higher initial scores display less pronounced growth rates. Note that the variance in test performance does not differ between Grades 2 and 4. Figures 1, 2 illustrate that growth rates were more pronounced in students from low-SES families who started with restricted comprehension scores in Grade 2 as well as for students from high-SES families who started with advanced comprehension scores. However, students from low-SES families did not exceed the competence level that medium- to high-SES students had in Grade 3, with students from high-SES families showing larger increases compared to students from medium-SES families across primary school. These results hint at an interesting pattern of the effects of schooling: While low-SES students now get access to presumably more complex language, a higher variety of connectives, and an environment that provides a wide range of explanations and various relations between arguments, resulting in an increase in comprehending connectives to the level already acquired by children from medium- to high-SES families in Grade 3, high-SES students now progress more compared to medium-SES students, learning even more complex connectives (e.g., *afterward, hence*) during primary school. Hence, our results show both a partial compensatory effect with respect to low- compared to middle-SES students with higher growth rates in low-SES students and a Matthew effect in students from high- compared to middle-SES families in acquiring more complex connectives with higher growth rates in high-SES students.

Our results regarding the development of the comprehension of connectives differ from findings of a general Matthew effect with higher-SES and majority language students progressing faster than lower-SES and language minority learners in academic language proficiency. Such a general Matthew effect has been documented for the development of other facets of academic language in primary school, particularly for listening comprehension at a text level (i.e., comprehension of orally presented texts characterized by typical features of academic language) as well as for general academic vocabulary (Volodina et al., 2020; Heppt and Stanat, in press). Our results also add to and partially differ from findings of previous studies on the comprehension of connectives, which demonstrated a general compensatory effect for language minority learners (e.g., Droop and Verhoeven, 2003; Crosson and Lesaux, 2013b). In accordance with the latter studies, we also find a compensatory effect for low-SES students, though not for students from medium-SES families. Note that contrary to these studies, our study considered monolingual and language minority learners from a broad range of SES backgrounds; this could – as Figure 2 illustrates – explain the findings and hints at the importance of considering SES. With regard to the findings on the development of other facets of academic language, we also find a Matthew effect for the comprehension of connectives in monolingual majority language students from high-SES families compared to the others. Yet, the negative correlation between the intercept and growth suggests that – at least partially – different mechanisms may account for the development of comprehending connectives. In our study, especially SES and students’ receptive grammar showed effects on both the intercept and the slope of the comprehension of connectives. Results of previous studies suggest that children’s receptive grammar is significantly predicted by the complexity of language input (Huttenlocher et al., 2002, 2010) and that the grammatical complexity of parents’ language mediates SES effects on grammar development in preschool children (Anderka, 2018). Advanced grammar skills in turn might help to bootstrap the meanings of more complex connectives. Students from low-SES families with presumably reduced access to more complex language and to a broad range of (more complex) connectives at home seem to progress faster and to profit more from schooling than students from medium-SES families, who start with an already advanced knowledge of more complex connectives in Grade 2. However, students with high-SES background outperform them in acquiring even more complex connectives – probably due to their advanced receptive grammar as well as to an enriched academic language environment at home.

Importantly, several connectives, such as *consequently, thus*, and *despite*, remain difficult even for students with high SES in Grade 4. Although some connectives seemed to be more challenging than others, these results should be treated with caution, as students’ responses may additionally depend on (the complexity of) the specific distractors as well as on specific sentence structures. In particular, in the German language, sentences with a verb in the second – as compared to a verb in a final – position may influence the ease of processing. In fact, descriptive analyses showed that connectives appearing in a sentence with a verb-final order were easier than those appearing in a sentence with a verb-second order. This may be due to differences in the processing load of different sentence structures (Bloem et al., 2017). The fact that, in the German language, connectives are related to the verb position within the sentence may partly explain the effect of receptive grammar on the comprehension of connectives that we found in our study. Furthermore, note that the scale on the comprehension of connectives used in our study included three distractors, two that were semantically incorrect and one that was semantically closer to the correct choice (but grammatically inadequate). Several other studies used tests on knowledge of connectives that included only grammatically (but not semantically) fitting foils (e.g., Welie et al., 2017; Kohnen and Retelsdorf, 2019). Across all grades, we found only a slightly higher preference for the distractors that were semantically related to the correct choice. In particular, monolingual German students had a higher frequency of selecting these distractors compared to language minority learners. These results hint at a tendency to concentrate on content, as also noted in a number of other studies (e.g., McClure and Geva, 1983).

Although we found an effect of receptive vocabulary on the comprehension of connectives in simple conditional models, contrary to other studies (e.g., Crosson et al., 2008), this effect disappeared in those models that considered all predictor variables. Note that we included indicators of both receptive vocabulary and receptive grammar (sentence comprehension) in these analyses, which – to the best of our knowledge – have not been considered before when studying factors associated with the comprehension of connectives, despite the interrelation between the processing of sentence structure and connectives. Furthermore, controversial findings may also result from sample differences [e.g., the sample used by Crosson et al. (2008) consisted of 90 language minority learners from low socioeconomic backgrounds, whereas our sample included both monolingual and language minority learners from various socioeconomic backgrounds], warranting further studies.

Our study is not free from limitations. Academic language is highly valued in schools and acquired through exposure (e.g., in written texts) and practice in academic discourses (Wong Fillmore and Snow, 2000). Unfortunately, we could not consider school opportunities to acquire the language of schooling (e.g., exposure to informational books at school) in our study. Furthermore, our assessment of the comprehension of connectives explicitly draws the students’ attention to the connectives and forces them to think about their meaning. This does not necessarily imply that these students actually use their knowledge in less reflexive situations (e.g., when following a discourse in school or when being presented with a sentence judgment task). In effect, a study of Dragon et al. (2015) hints at the fact that primary school children often ignore more complex connectives and just stick to an overall scenario-based comprehension of the connected sentences. In particular, the simultaneous examination of the comprehension and production of connectives in different task contexts seems to be an interesting topic for future studies. Finally, it is important to note that, in Germany, language minority students are a heterogeneous group – especially with regard to the conditions and pathways of acquiring the language of the majority. Some of them acquire both the minority and the majority language from the very beginning and their parents are native speakers of at least one of the languages, or they learn it from their parents who themselves are not highly proficient in German; others may start to learn German in kindergarten or even later. This issue should be considered when interpreting our results with regard to language minority learners.

In our study, we analyzed the impact of various background factors on the development of academic language across primary school age. As we found a gap between SES groups as well as between German monolinguals and language minority learners in Grade 2, one promising area of research is to investigate the specific features of the home learning environment that might impact the development of connective comprehension at preschool and early school age. In our analyses, we used a scale on joint preschool activities of parents with their children. However, this scale mainly included home literacy activities. As already mentioned, other studies hint at the fact that these are particularly relevant for learning to read, whereas the complexity of parents’ grammar seems to be especially relevant to grammar acquisition (Anderka, 2018; see also Lehrl et al., 2012). In addition, providing children with explanations and in-depth talk on specific subjects might be particularly relevant to the acquisition of connectives. For instance, Napoli and Purpura (2018) suggested that parent–child numeracy practices may present children with in-depth verbal interactions and explanations that could contribute to their language learning. In general, when studying the development of the comprehension of connectives at preschool age and at school, an in-depth focus on developmental pathways in students from various SES backgrounds seems to be needed. The principle of environmental specificity suggests that various aspects of development are characterized by unique environmental predictors (Wachs and Chan, 1986). Thus, SES may affect child development through multiple, specific pathways, with each path being a reflection of specific proximal processes (Bronfenbrenner and Morris, 1998) by which environmental factors exert their effects. Though our study shows receptive grammar to be especially relevant to SES differences in comprehension of connectives and its development, different mediators between SES and the acquisition of connectives should be addressed more explicitly in future research.

Furthermore, amongst others, connectives have been suggested to be especially prevalent and crucial to support the communication of information across content areas (see Silliman et al., 2018, for an overview). Drawing on a sample of native French-speaking university students learning English, Geva (1992) showed that the ability to construct coherence relations at the level of clauses and sentences is a necessary precursor to constructing such coherence relations in extended discourse. Cain and Nash (2011) reported that young readers (8- and 10-years-old) have an adequate ability to understand various connectives and are able to take advantage of information provided by connectives as they read. However, Cain and Nash (2011) also found that the explicit understanding of 8- and 10-years-old children appears to lag behind their ability to benefit from these signals when reading, which may affect other school tasks (e.g., written text production). These findings suggest that the role of the comprehension of connectives for, for example, various achievement measures may be a promising research area. In effect, recent studies show that the comprehension of connectives is related to performance on various achievement measures in reading and in mathematics in primary school (e.g., Volodina et al., unpublished).

As to practical implications, for example, the creation of various instructional and learning environments that promote the development of academic language skills of students could be considered (Roessingh et al., 2005; Cummins, 2008). Duke (2000) pointed out the paucity of informational texts in the early grades, amongst others, in classroom (written) language activities and noted that informational texts were particularly scarce in the classrooms in low-SES settings. *Instructional conversations* that focus on the language used in the materials with which students are confronted at school are considered to be useful for improving the academic language skills (Wong Fillmore, 2009) of students from various socioeconomic backgrounds. As Langer (2015) pointed out, academic language should be taught “through first-hand disciplinary experiences, as language and thought-in-use, in content-area classes” (p. 4).

## Author’s Note

The study uses items from Heppt et al. (2020). BiSpra 2–4. Test zur Erfassung bildungssprachlicher Kompetenzen bei Grundschulkindern der Jahrgangsstufen 2 bis 4 (BiSpra 2–4. Test to measure academic language competences of primary school children in Grades 2–4). AV and SW are two of the co-authors of the BiSpra 2-4 test. Copyright is by Waxmann publishing house.

## Data Availability Statement

Publicly available datasets were analyzed in this study. This data can be found here: https://www.iqb.hu-berlin.de/fdz/studies/BiSpra_2.

## Ethics Statement

This study was carried out in accordance with the APA ethical guidelines. Written informed consent to participate in this study was provided by the participants’ legal guardian/next of kin. The Ethics Committee of the University of Bamberg has reviewed and approved the BiSpra II project.

## Author Contributions

AV and SW contributed to the theoretical conception of the study and the interpretation of the results. AV performed the statistical analyses and drafted the manuscript. SW reviewed and edited the manuscript. Both authors have made a substantial intellectual contribution to the manuscript and approved it for publication.

## Conflict of Interest

The authors declare that the research was conducted in the absence of any commercial or financial relationships that could be construed as a potential conflict of interest.

## Footnotes

1 Similar statistical procedures were used by Volodina et al. (2020), when analyzing the development of general academic vocabulary.

2 Please note that the pattern of results reported does not differ from the pattern of results obtained without multiple imputation.
